# Iatrogenic instability of the acromioclavicular joint leads to ongoing impairment of shoulder function even following secondary surgical stabilization

**DOI:** 10.1007/s00402-022-04387-4

**Published:** 2022-02-27

**Authors:** Stephanie Geyer, Andrea E. Achtnich, Andreas Voss, Daniel P. Berthold, Patricia M. Lutz, Andreas B. Imhoff, Frank Martetschläger

**Affiliations:** 1 Department for Orthopaedic Sports Medicine, Technical University Munich, Klinikum Rechts Der Isar, Munich, Germany; 2 Department of Trauma Surgery, University Medical Center, Regensburg, Germany; 3 Deutsches Schulterzentrum, ATOS Klinik München, Effnerstr. 38, 81925 Munich, Germany; 4 Sporthopaedicum, Regensburg, Germany

**Keywords:** Acromioclavicular joint, AC joint, Revision, Iatrogenic, AC joint instability, Lateral clavicular resection

## Abstract

**Purpose:**

Iatrogenic instability of the acromioclavicular joint (ACJ) following distal clavicle excision (DCE) represents an infrequent pathology. Revision surgery to restore ACJ stability and alleviate concomitant pain is challenging due to altered anatomic relationships. The purpose of this study was to evaluate the used salvage techniques and postoperative functional and radiological outcomes in retrospectively identify patients with a painful ACJ following DCE. We hypothesized that iatrogenic instability leads to ongoing impairment of shoulder function despite secondary surgical stabilization.

**Methods:**

9 patients with a painful ACJ after DCE (6 men, 3 women, 43.3 ± 9.4 years) were followed up at a minimum of 36 months after revision surgery. Besides range of motion (ROM), strength and function were evaluated with validated evaluation tools including the Constant score and the DASH score (Disability of the Arm, Shoulder and Hand questionnaire), specific AC Score (SACS), Nottingham Clavicle Score (NCS), Taft score and Acromioclavicular Joint Instability Score (AJI). Additionally, postoperative X-rays were compared to the unaffected side, measuring the coracoclavicular (CC) and acromioclavicular (AC) distance.

**Results:**

At follow-up survey (55.8 ± 18.8 months) all patients but one demonstrated clinical ACJ stability after arthroscopically assisted anatomical ACJ reconstruction with an autologous hamstring graft. Reconstruction techniques were dependent on the direction of instability. The functional results demonstrated moderate shoulder and ACJ scores with a Constant Score of 77.3 ± 15.4, DASH-score of 51.2 ± 23.4, SACS 32.6 ± 23.8, NCS 77.8 ± 14.2, AJI 75 ± 14.7 points and Taft Score 7.6 ± 3.4 points. All patients stated they would undergo the revision surgery again. Mean postoperative CC-distance (8.3 ± 2.8 mm) did not differ significantly from the contralateral side (8.5 ± 1.6 mm) (*p* > 0,05). However, the mean AC distance was significantly greater with 16.5 ± 5.8 mm compared to the contralateral side (3.5 ± 1.9 mm) (*p* = 0.012).

**Conclusion:**

Symptomatic iatrogenic ACJ instability following DCE is rare. Arthroscopically assisted revision surgery with an autologous hamstring graft improved ACJ stability in eight out of nine cases (88.9%). However, the functional scores showed ongoing impairment of shoulder function and a relatively high overall complication rate (33.3%). Therefore, this study underlines the importance of precise preoperative indication and planning and, especially, the preservation of ACJ stability when performing AC joint resection procedures.

**Level of Evidence:**

Case series, LEVEL IV.

## Introduction

Revision acromioclavicular joint (ACJ) surgery remains challenging, irrespective of the initial operation. Alterations of stabilizing bony and ligamentous structures of the ACJ can hereby complicate adequate restoration of native ACJ stability and subsequent shoulder function. Currently, more than 150 procedures for ACJ reconstruction have been described, which emphasizes the incertitude how to best treat this challenging patient cohort. [1] Consequently, complication rates are often dependent on the type of repair and high surgical learning curves, with failure rates ranging from 20—88.9%. [19–21, 24, 25, 33] A recent systematic review highlights the importance of restoring native biomechanical properties in revision ACJ surgery. [11] Dyrna and colleagues introduced four different failure categories based on the failure mechanism and subsequent surgical approach including failure of treatment combined with excessive bone loss at the distal clavicle (distal clavicle excision; DCE). [11] Thus, a detailed failure analysis in revision ACJ surgery is of great importance, as the impact of previous ACJ stabilizations on postoperative outcomes in revision surgery still arises concern among shoulder surgeons. This may be especially the case, when bony disorders such as bone loss at the distal clavicle or large bone tunnels created in the clavicle or/and coracoid are encountered. Unfortunately, inadequate resection such as under- or overresection of the distal clavicle may result in significant pain or recurrent ACJ instability. Biomechanically, Beitzel et al. showed that a DCE resection over 10 mm leads to an increased anterior translation, even if the acromioclavicular (AC) capsule and coracoclavicular (CC) ligaments remain untouched. [3] This was also shown by Böhm et al., who underlines the potential danger of iatrogenic ACJ instability following DCE. [7] In contrast, Blazar et al. reported a greater CC-distance in stress radiographs compared to the unaffected shoulder in 17 patients after isolated DCE, without having any clinical relevance. [6] To this, secondary ACJ instability following ACJ resection is often not explicitly mentioned. [26, 27] Consequently, secondary iatrogenic instability after DCE is a rare condition with underwhelming high-level evidence. As such, decision-making and optimal treatment choice remains challenging for orthopedic surgeons in daily clinical practice.

The purpose of this study was (1) to report the surgical revision techniques (2) and subsequent clinical and radiographic outcomes in patients treated after iatrogenic instability of the ACJ following DCE. The initial pathology and salvage procedures, as well as associated functional, cosmetic and radiographic outcomes were described and evaluated. The authors hypothesized that iatrogenic ACJ instability due to DCE would have a persistent negative impact on shoulder function, even following revision surgery.

## Material and methods

### Patient selection

Institutional review board approval was obtained prior to initiation of the study ((233/14)) and the study was conducted according to the Declaration of Helsinki. A retrospective chart review was performed on patients undergoing revision ACJ surgery following DCE at the author’s institution between October 2012 and December 2016. Patients eligible for study inclusion were those aged ≥ 18 years who underwent revision ACJ surgery and had previously DCE. Indication for revision surgery included persisting pain for at least 5 months due to clinically and radiographically evident ACJ instability. Patients with relevant concomitant injuries such as fractures of the shoulder girdle, nerve or vessel lesions, any rheumatic disease and any prior traumatic event of the contralateral non-affected shoulder/ACJ, were excluded from the study. Demographic data including age, sex, hand dominance and previous nonoperative and operative treatment were collected. Surgical charts were analyzed for additional intraarticular pathologies, surgical technique including previously used hardware and graft, intraoperative complications and postoperative protocol. Informed consent was obtained by each individual prior to the clinical and radiological evaluation.

### Functional and clinical outcomes

At final follow-up, ACJ instability was assessed by a fellowship trained shoulder surgeon (F.M.) for horizontal and vertical direction. Patients underwent a thorough clinical examination with a goniometer to evaluate range of motion (ROM). The abduction strength was measured using an IsoBex® (MDS Medical Device Solutions AG, Oberburg, Switzerland) in comparison to the contralateral side.

The primary outcome scores were the Constant score, the DASH score (Disability of the Arm, Shoulder and Hand questionnaire), and specific ACJ questionnaires (AC Score (SACS), Nottingham Clavicle Score (NCS), Taft score and Acromioclavicular Joint Instability Score (AJI)). Patients were asked for subjective evaluations of the postoperative outcome with the subjective shoulder value in % (SEV) and visual satisfactions scale (from 1–10 points). These scores were collected at a minimum of 36 months postoperatively. Furthermore, the postoperative return-to-sport rate and the sporting activity level were evaluated. Complications such as infections, nerve lesions or nerve syndromes, stiffness, persistent instability and surgical revisions were collected.

### Radiographic analysis

Standardized radiographs (bilateral panorama view; modified y-view (Alexander view) of the ipsi- and contralateral side) were taken at final follow-up. The coracoclavicular (CC) distance, acromioclavicular distance (AC), clavicular length (CL) and bone loss of the lateral clavicle (ΔCL = unaffected – affected side) were measured pre- and postoperatively in mm by two fellowship trained shoulder surgeon (F.M. and A.A.) and compared to the contralateral side. Using the bilateral panorama view, CC distances for each ACJ (both affected and non-affected) were determined by measuring the shortest distance in mm from the most superior point of the coracoid process to the nearest point of the distal clavicle and for AC distances from the most lateral point of the clavicle to the most medial point of the acromion in mm [5, 30]. For CL the center point of the medial and lateral ending of the clavicle was defined and the length of the line between the two points was measured in mm. All measurements were taken twice at intervals of 2 weeks for inter- and intra-reliability testing.

### Surgical technique for revision ACJ surgery

Arthroscopically assisted ACJ stabilization procedures with ipsilateral hamstring tendon graft were used as salvage procedure. One possible technique using a gracilis or semitendinosus tendon autograft with bicortical fixation using an endobutton and high-tensile fiber tapes was previously described by the senior author (F.M.) for chronic ACJ instability. [23] As excessive bone loss of the distal clavicle may occur, correct and safe placement within the safer zone described by Voss et al. and Geaney et al. was noted to be challenging. As significant bone loss at the distal clavicle site was shown to be highly associated with recurrent horizontal and rotational instability of the ACJ rather than vertical instability, two surgical techniques were used depending on the kind of instability. Modifications of the surgical technique are shown in Table 1.

**Table 1:** Initial pathology, surgical technique and complications

No.	Initial pathology	Prior surgeries	Arthroscopically assisted surgical technique	Complications
1	Rockwood IV	ACJ Stabilization with Balser plate 04/04, ME&DCR 04/04	AC-reco with gracilis	
2	ACJ arthritis	06/11 ARAC, 10/11 Weaver–Dunn plus DCR	CC-reco with Tightrope and gracilis	Coracoid fracture, temporary K-wire fixation and revision with hook plate
3	Tuberculum majus#, ACJ arthritis	ORIF Tuberculum majus and ME, 02/13 & 06/13 ARAC	AC-reco with gracilis	
4	ACJ arthritis	03/12 & 06/14 ARAC	CC-reco with Fibertape plus 2 × Dogbone, AC reco with gracilis	
5	ACJ arthritis	08/13 ARAC	CC-reco with Fibertape plus 2 × Dogbone, AC reco with gracilis	
6	Rockwood V	01/14 ORIF Hook plate; 02/14 ME, 11/14 ARAC	CC-reco with Fibertape plus 2 × Dogbone & gracilis, AC reco with gracilis	Dislocation of subcoracoidal dog bone
7	ACJ arthritis	2/12 ARAC, 02/14 LBS TD, SSC release, ARAC	CC-reco with Fibertape plus 2 × Dogbone, AC reco with SemiT	
8	ACJ arthritis	1/12, 8/12 ARAC, 2/14 AC stabilization with Fibertape	AC reco with Fibertape plus SemiT	Granuloma due to sutures, revision with excision of the granuloma
9	ACJ arthritis	9/13 ARAC	AC-reco with gracilis	

*f* female, *m* male, *ACJ* acromioclavicular joint, *ME* material excision, *DCR* distal clavicle resection, *AS* arthroscopy, *AC-reco* acromioclavicular reconstruction, *Gracilis* gracilis tendon, *SemiT* semitendinosus tendon, *ARAC* arthroscopical resection of the acromioclavicular joint, *ORIF* open reposition and internal fixation

### Horizontal instability—AC reconstruction with ipsilateral gracilis graft

**Fig. 1:**
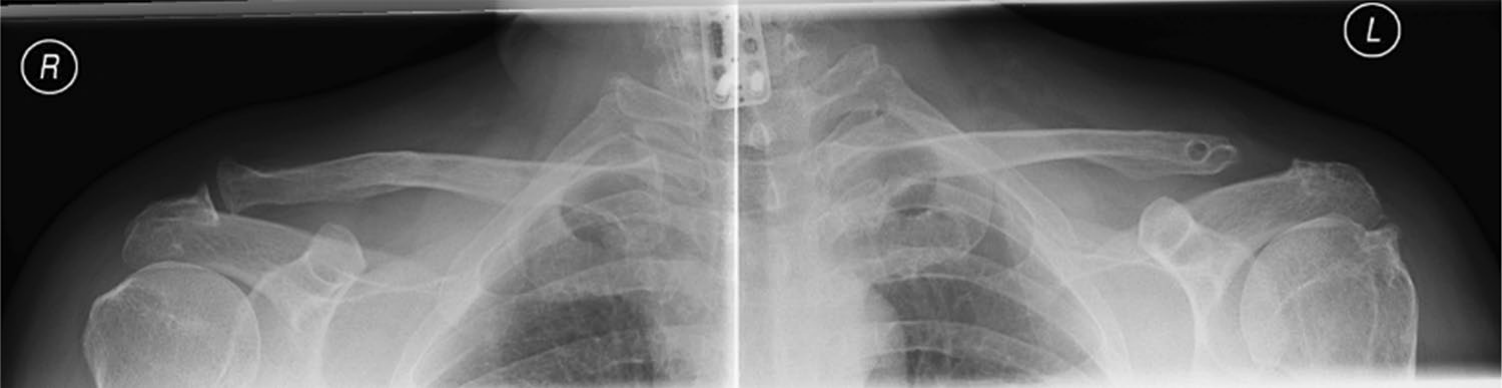
Acromioclavicular reconstruction (left side) of an iatrogenic, horizontal instability of the acromioclavicular joint with the ipsilateral gracilis tendon graft

With the patient in the beach chair position and the arm engaged in a hydraulic arm holder, the ipsilateral gracilis tendon (*n* = 7) or semitendinosus tendon graft (*n* = 2) was harvested with a tendon stripper in standardized fashion. The graft was used in full length (approximately 20 cm) and both sides were prepared using torpedo-like stitches (e.g., Vicryl 2.0). A standard posterior arthroscopic portal was established, followed by diagnostic arthroscopy of the shoulder joint. Concomitant pathologies were addressed first, if necessary. Three patients showed concomitant SLAP lesions of the long head of the biceps tendon, which were treated with either tenotomy (1) or subpectoral tenodesis (2). Next, a 4- to 5-cm-long incision was made in line with the distal clavicle followed by incision of the deltotrapezial fascia. A 4-mm drill hole in the distal clavicle was established by placing first a K-wire from anterior to posterior 1 cm from the distal boarder, which was then over-drilled. Similarly, a second drill hole was placed through the acromion (Fig. 1). Subsequently, the tendon graft along with a fiber tape (Arthrex Inc., Naples, FL, USA) was shuttled in the figure-of-eight technique and knotted in the mid portion of the ACJ. Alternatively, the fiber tape (Arthrex Inc., Naples, FL, USA) was shuttled in a box technique while the tendon was shuttled in a figure-of-eight technique by using the shuttle sutures (Arthrex Inc., Naples, FL, USA). The fiber tapes (Arthrex Inc., Naples, FL, USA) and the graft were then knotted and the loose ends of the graft were used as an interposition in-between the ACJ. Care was taken to meticulously close the deltotrapezial fascia.

### Horizontal and vertical instability—AC reconstruction with ipsilateral gracilis tendon graft and CC reconstruction using high-tensile fibertapes and dogbones

**Fig. 2:**
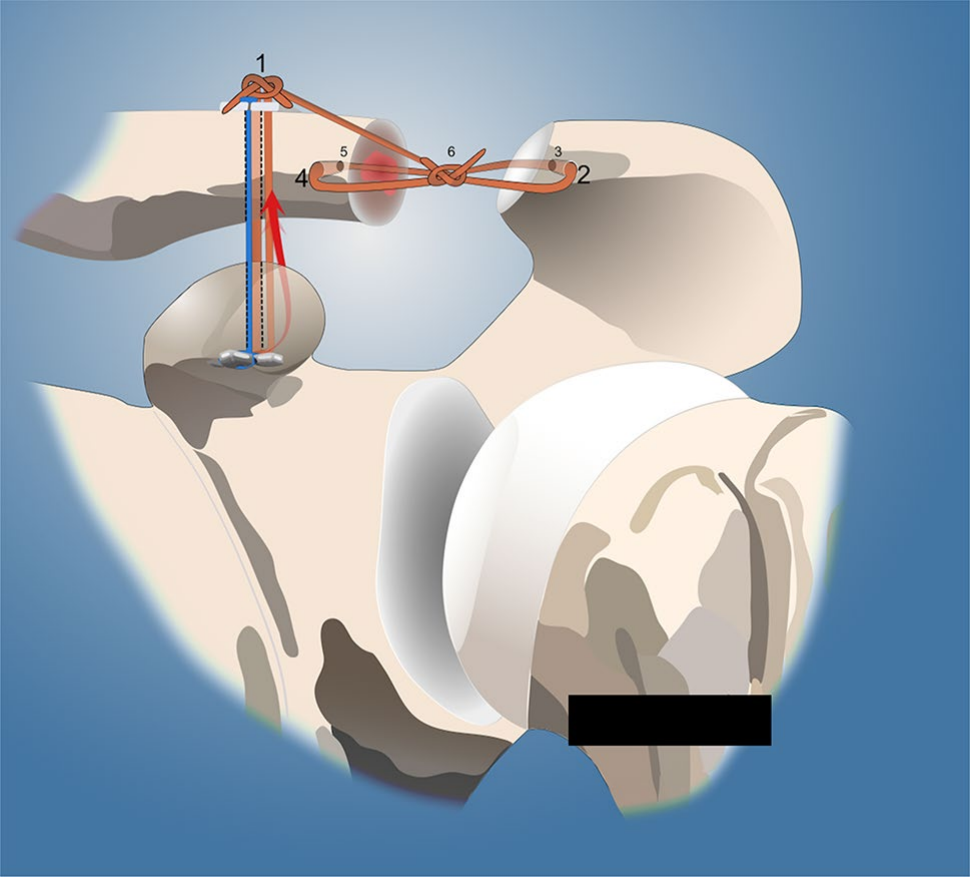
Coracoclavicular (CC) reconstruction with autologous ipsilateral gracilis graft with an additional suture tape and acromioclavicular (AC) reconstruction with the longer limb of the graft (blue filled rectangular) suture tape (red filled rectangular) gracilis graft (arrow direction) direction of passage of the graft behind the coracoid process and anteriorly to the clavicle; 1: knotting and sewing the graft at the clavicle, 2/3: passing the longer limb from anterior to posterior through the acromion, 4/5: passing it through the lateral clavicle from anterior to posterior, 6: knotting and sewing it as interposition in-between the acromioclavicular joint (ACJ)

**Fig. 3:**
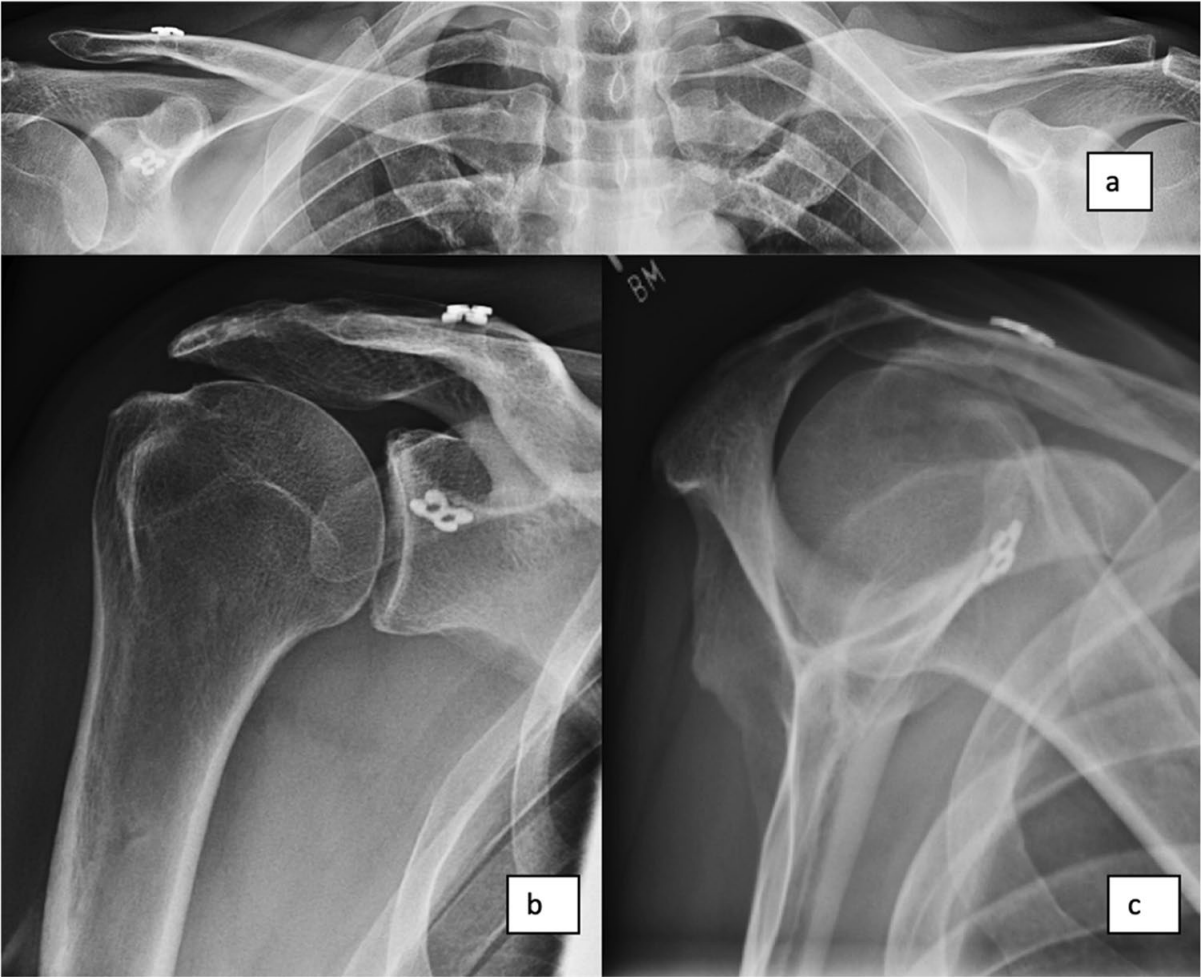
Follow-up X-ray after acromioclavicular (AC) reconstruction with ipsilateral gracilis graft and coracoclavicular (CC) reconstruction with Fibertapes and 2 × Dogbones (Arthrex Inc., Naples, FL, USA); **a** panorama view, **b** a.p. view and **c** Alexander view

The placement of the patient, harvesting of the tendon and diagnostic arthroscopy was performed in the above-mentioned manner. Via a prior established anterolateral portal, the arthroscope was switched and the base of coracoid was explored and skeletonized with a radio-frequency device. Again, a 4- to 5-cm-long incision was made followed by incision of the deltotrapezial fascia and exposure of the ACJ and distal clavicle. For CC-fixation a K-wire was placed through the distal clavicle to the center of the base of the coracoid. The K-wire was when over-drilled with a 4-mm cannulated drill. At the distal clavicle, a horizontal bone tunnel of 4-mm was established from anterior to posterior. Equally, a 4-mm bone tunnel was created at the anterior portion of the acromion. With the use of a suture passing device (Arthrex Inc., Naples, FL, USA) the fiber tape, the cortical fixation button (DogBone, Arthrex Inc., Naples, FL, USA) and the free end of the graft were placed in the bone tunnels of the clavicle and coracoid from inferior to superior, respectively. Under arthroscopic view, the fixation button then was placed above the graft at the coracoid base. The free end of the graft was then retrieved anteriorly to the clavicle using a grasper. Reduction of the ACJ joint was fluoroscopically verified and the fiber tape was knotted using a second cortical fixation button at the lateral clavicle. Finally, the graft was then knotted and the loose end of the graft was used for subsequent AC reconstruction by pulling the graft through the bone tunnels of the lateral clavicle and the acromion. The loose ends were tightened and sutured with a Fiberwire No. 2 (Arthrex Inc., Naples, FL, USA). Again, reconstruction of the deltotrapezial fascia was performed meticulously (Figs. 2, 3).

### Postoperative rehabilitation

Postoperative rehabilitation consisted of wearing a shoulder sling (Medi, Bayreuth, Germany) for 6 weeks. Passive abduction and flexion were limited to 30° and internal/external rotation was limited to 80°/0/30° during the first two weeks. Abduction and flexion were increased to 45° active-assistive motion during week 3 and 4. After 5 weeks postoperatively, active abduction and flexion until 60° were accomplished with free internal and external rotation. 6 weeks postoperatively, free active-range of motion was allowed while return-to-work and return-to-sports were expected 12 weeks postoperatively.

### Statistical analysis

Descriptive statistics including mean, minimum, maximum and standard deviations were calculated for continuous variables. Categorical variables were examined and frequencies and percentages were documented. Inter- and intraclass correlation coefficient was determined for the AC, CC and CL measurements according to Koo and Li [16]. The Wilcoxon ranked-sign test was used for evaluation of statistical differences in terms of strength, AC, CC and clavicular distance and ACJI Score compared to the contralateral side as well as for passive glenohumeral range-of-motion (ROM). All analyses were performed with SPSS Statistics (IBM Corp. Released 2017, Version 25.0. Armonk, NY, USA). The alpha level for all analyses was set at 0.05.

## Results

In a single surgeon’s practice, 11 patients underwent revision ACJ reconstruction using a tendon allograft between October 2012 to December 2016. Of these patients, 9 patients (6 men; 3 women) were eligible for inclusion in the study (Table 1).

Mean age of patients was 43.3 ± 9.4 years. Surgery was performed in 6 left shoulders and 3 right shoulders. The dominant arm was affected in 44.4% of the patients. The initial diagnosis leading to primary ACJ surgery was ACJ dislocation (Rockwood type IV and V) in 2 patients and ACJ osteoarthritis in 7 patients, which was treated using DCE. The median time between primary surgery and the salvage (e.g., revision) surgery was 22.4 ± 26.9 months. The included patients were noted to have 2.3 ± 0.9 prior surgeries of the affected side.

Preoperatively, all patients reported pain and tenderness at the ACJ with positive clinical ACJ tests; *n* = 3 reported a feeling of recurrent ACJ instability; and *n* = 3 complained about painful instability leading to revision surgery. All 9 patients had documented clinical ACJ instability in anterior to posterior direction, while 3 patients showed additional vertical instability.

### Functional outcomes

At final follow-up (55.8 ± 18.8 months) 8 patients achieved clinical stability of the ACJ.

There was no significant difference in passive glenohumeral ROM between the affected and the non-affected side (*p* < 0.05). The abduction strength measured using the IsoBex® (MDS Medical Device Solutions AG, Oberburg, Switzerland) showed significantly lower strength for the affected shoulder, when compared to the non-affected side (4.5 ± 3.5 N vs. 7.9 ± 3.4 N (*p* = 0.012)).

### Radiological results

The radiological analysis is summarized in Table 2. Mean postoperative CC-distance did not differ significantly compared to the contralateral side (*p* > 0.05), but reached statistical difference compared from pre- to postoperative (*p* = 0.012). The AC distance of the affected side showed a significantly greater distance when compared to the contralateral side (*p* = 0.012). However, this did not reach statistical difference (*p* > 0.05) when compared to the preoperative status. The mean bone loss of the lateral clavicle was 12.3 ± 7.7 mm and the CL distance differed significantly compared to the unaffected side (*p* = 0.012). The interclass correlation coefficient was noted be excellent (1.0) according to Koo and Li ([16]. There was no statistically significant difference between baseline measurement and two weeks within the raters (RMANOVA time*rater *p*-value = 0.053).

**Table 2:** Radiological results

	Distance ± SD in mm			*P*-value
	Preoperative	Postoperative	Contralateral side ^1^	
Coracoclavicular distance	10.6 ± 3.2	8.3 ± 2.8	8.4 ± 1.6	0.012*
				0.866
Acromioclavicular distance	16.9 ± 6.3	16.5 ± 5.8	3.5 ± 1.9	0.779*
				**0.012**
Clavicular length	n.n	139.5 ± 15.5	151.7 ± 10.9	n.n
				**0.012**

Bold indicates significance (*P* < 0.05)

*mm* millimeter, ^1^all contralateral joint measurements were taken postoperatively, *comparison of the pre- and postoperative affected side, *n.n*. not noted

### Functional and clinical outcome scores

At final follow-up, the Constant Score was 77.3 ± 15.4 and the mean DASH-score 51.2 ± 23.4. However, the specific ACJ scores showed only moderate results: SACS 32.6 ± 23.8, NCS 77.8 ± 14.2 and Taft Score 7.6 ± 3.4 points. When compared to the unaffected side, the ACJI showed a significantly better score when compared to the operated side (96.9 ± 6.1 points vs. 75 ± 14.7 points) (p = 0.012).

### Patient rated outcomes

Overall, 9/9 patients reported that they would consider the same revision procedure again. All 9 patients felt an overall improvement of their shoulder function of 58.8% ± 19.6%. Postoperatively, a SEV of 68.8% ± 16.4% and 8 ± 2 points on VSC were achieved. At final follow-up, 88.9% of the patients returned to work while 55.5% of the patients returned to sports with a sportive activity level of 65.6%.

### Complications

Postoperatively, 3 patients showed complications which are summarized in Table 1. 1 patient suffered from a suture granuloma and had revision surgery requiring debridement after one month. One patient suffered from a coracoid insufficiency fracture and received revision surgery with autograft and hook plate. After removal of the hardware, the patient demonstrated a stable ACJ with reduced pain. One patient suffered secondary dislocation of the subcoracoidal implant but denied any revision surgery as good overall shoulder function was noted. No patient complained about graft donor site-related issues at the knee.

## Discussion

The most important finding of this study was that restoring stability of the ACJ using the presented arthroscopic assisted ACJ stabilization in patients with iatrogenic ACJ instability due to DCE leads to good functional and clinical outcomes, however, ongoing impairment of shoulder function with a high complication rate of 33% may be expected. More importantly, all patients included, would undergo the same surgical procedure again, as it was noted to significantly reduce the impairment when compared to the preoperative status. As such, a subjective shoulder function improvement of 58.8% with a return-to-work rate of 88.9% and return-to-sport rate of 55.5% was reached at final follow-up.

In current orthopedic literature, a variety of salvage procedures for recurrent ACJ instability have been described with respect to the initial diagnosis and failure mechanism. [13–15, 29] Of interest, the majority of the studies are hereby reporting on recurrent ACJ instability mostly due to loss of reduction, which was noted to be either associated with or without an adequate re-trauma. [29] Consequently, only a limited number of case reports currently exist reporting on revision surgeries following DCE, mostly including soft tissue reconstruction techniques similar to the original Weaver and Dunn technique. [32] Recently, Tauber et al. published a case series of 12 patients with various and multiple previous surgeries including DCE, modified Weaver Dunn procedures, removal of heterotopic calcifications as well as Bosworth screw fixation and tension band wiring. [29] However, the authors did not specify how many of these patients suffered from iatrogenic caused recurrent instability of the ACJ. In contrast to the technique presented in this manuscript, patients were revised with ipsilateral semitendinosus reconstruction by sole slinging of the tendon graft around the coracoid and through two separate bone tunnels through the clavicle. For additional graft protection, cerclage wires were used in 10 patients, while 2 patients required Bosworth screw fixation. However, AC instability was not separately addressed, even though 40% of the patients showed posterior translation of the distal clavicle on preoperative axillary X-ray view. The mean postoperative Constant score was 76.4 points (range 46–91), which is similar to the outcome scores of the present series. However, this should still be considered as moderate, emphasizing that major improvements are needed in order to restore the native biomechanical properties of the ACJ.

Additionally, the radiological analysis showed a significant decrease of the CC-distance compared to the preoperative measurements and an unchanged mean AC distance of 15 mm (range 11-39 mm). Of interest, significantly decreased posterior displacement of the distal clavicle in the axial view compared to preoperatively was noted. However, the overall complication rate was only 16.7%. Similarly, LaPrade and Hilger used a semitendinosus graft technique for open CC reconstruction, passing it in a loop configuration through one drill hole through the coracoid process and one drill hole through the clavicle (and suturing it together) in two patients following failure after modified Weaver–Dunn procedure [18]. Again, additional AC stabilization was not performed and neither shoulder- nor specific AJC scores have been evaluated postoperatively. Interestingly, the aforementioned case series rather focus on isolated CC reconstruction without paying special attention to the importance of the AC capsule and ligaments.

Recently, the biomechanical importance of an intact AC joint capsule and restoring both the AC and CC ligamentous complex for ACJ stability has been highlighted by several authors. [2, 28]. As such, the hazard of iatrogenic recurrent ACJ instability following DCE has gained popularity among shoulder surgeons as overresection often comes along with persistent horizontal and vertical instability resulting in significant shoulder pain and impairment. [3, 7]. In an anatomical study by Boehm et al., a DCE of less than 1 cm is highly recommended to protect the CC ligaments and preserve ACJ stability. [7] Of interest, in the present study, a loss of 12.3 ± 7.7 mm of the distal clavicle led to recurrent horizontal instability in 66.7% and to a combined vertical and horizontal ACJ instability in 33.3% of the cases. The authors therefore support the findings of Branch et al. who stated that a resection of distal clavicle of 5 mm is sufficient to ensure no bone contact and to simultaneously preserve ACJ stability. [8] Resections of greater than 8 mm (women) or 10 mm (men) may put the trapezoid ligament at risk. Understanding the ligamentous and bony stability complex of the ACJ rationalizes the aforementioned cases of extensive joint instability and pain leading to an almost complete loss of shoulder function.

When approaching revision ACJ surgery, several techniques using all kind of grafts or hardware have been proposed. Carofino et al. introduced a novel open surgical technique to address horizontal ACJ instability by using the longer limb of the already passed graft for CC stabilization and by looping it over the AC joint. Fixation is then performed using a high-tensile transosseous suture to the posterior joint part of the acromion, including the trapezial fascia and AC ligament and passing it in mattress configuration to the anterior part. [10] Biomechanically, Beitzel et al. showed that rotational and translational stability of the ACJ was restored best by suturing and direct wrapping when compared to transacromial techniques. [2] Dyrna et al. described that placing transacromial tunnels did not result in an increased risk of fracture. [12] To prevent iatrogenic acromial fractures, positioning of the bone tunnel should be performed at the anterior half of acromion by using the smallest bone tunnel diameter needed to ensure optimal fixation [12, 31]. This was confirmed by Martetschläger et al. in a biomechanical study, whereas drill holes with a diameter greater than 4 mm for CC reconstruction are more likely to be associated with a coracoid fracture than smaller drill holes (e.g., 2.4 mm). [22] In the present study, AC stability was restored by passing the longer limb of the graft through a 4-mm horizontal bone tunnel at the distal clavicle and the anterior part of the acromion. Postoperatively, no peri-implant fracture within the clavicle or the acromion was found. However, in the present case series, one patient showed an insufficiency fracture of the coracoid process leading to re-instability. Interestingly, this patient was the only one who had received a CC stabilization procedure without addressing the horizontal instability. Overall, blowout fractures of the coracoid after ACJ stabilization remain a rare disease, which was also confirmed by Thangaraju et al., who published 4 cases with fracture after ACJ stabilization procedure. [30] Consequently, only one patient showed a fracture of the coracoid base which was treated conservatively.

At a final point, the data gathered from this study underline that care should be taken to prevent bony disorders such as bone loss at the distal clavicle or large bone tunnels created in the clavicle or/and coracoid. The precise amount of recommended resection length at the distal clavicle remains unknown, however may not exceed the 5 mm recommend by the authors. In the past ten years, surgical techniques to address the ACJ in the acute and chronic condition have continuously evolved by introducing arthroscopically assisted procedures and becoming much less invasive. [9, 23] The authors believe that especially revision surgeries may benefit from an arthroscopic approach. From the subacromial view, instability of the ACJ and the amount of bone resection at the distal clavicle can be confirmed under direct visualization and concomitant pathologies such as SLAP lesions or biceps tendinitis may be addressed in the same surgery. Furthermore, arthroscopic visualization enables safe placement of the coracoid button at the base of the coracoid process.

There were several limitations to this study. The limitations of the present case series include the retrospective nature with a limited number of patients and heterogenic preoperative patients’ history. However, the number of patients is limited due to the rare nature of the pathology itself. To this, current literature investigating revision ACJ surgery reported on smaller or similar patient cohorts. [4, 17, 29] Given the retrospective character of the study, there were no preoperative functional scores for comparison to the postoperative result. Also, the series may be underpowered to draw a definitive conclusion about which fixation method is preferable and if age, number of prior surgeries, manner of prior surgery and mm of excision of the lateral clavicle have an influence on the postoperative result. As such, all observations may be limited to a type II error due to the small patient cohort. Another limitation was that two surgical techniques were used depending on the kind of instability: moreover, smaller modifications such variations of AC reconstruction (box technique vs. figure of eight) or the use of a different graft (semitendinosus tendon) confound comparability.

## Conclusion

The presented arthroscopic assisted ACJ stabilization technique in patients with iatrogenic vertical and/or horizontal instability due to DCE leads to good functional and clinical outcomes, however, ongoing impairment of shoulder function with a high complication rate of 33% may be expected. More importantly, all patients included would undergo the same surgical procedure again, as it was noted to significantly reduce the impairment when compared to the preoperative status. As such, a subjective shoulder function improvement of 58.8% with a return-to-work rate of 88.9% and return-to-sport rate of 55.5% was reached at the final follow-up.

Compared to the good and excellent results known after acute or chronic AC joint stabilization procedures, the postoperative clinical outcomes following stabilization procedures after DCR remain weak. Therefore, this study underlines the importance of a thorough knowledge about the complex ligamentous and bony stability of the ACJ before performing any ACJ surgery in order to avoid these difficult-to-treat cases in the future.
